# Common findings on head computed tomography in neonates with confirmed congenital Zika syndrome

**DOI:** 10.1590/0100-3984.2017.0119

**Published:** 2018

**Authors:** Natacha Calheiros de Lima Petribu, Andrezza Christine Vieira Fernandes, Marília de Brito Abath, Luziany Carvalho Araújo, Felipe Reis Silva de Queiroz, Janniê de Miranda Araújo, Glauber Barbosa de Carvalho, Vanessa van der Linden

**Affiliations:** 1 Hospital Barão de Lucena (HBL) and Instituto de Medicina Integral Prof. Fernando Figueira (IMIP), Recife, PE, Brazil.; 2 Hospital Barão de Lucena (HBL), Recife, PE, Brazil.; 3 Clínica Imago, Campina Grande, PB, Brazil.

**Keywords:** Skull, Tomography, X-ray computed, Zika virus, Communicable diseases, emerging, Infant, newborn

## Abstract

**Objective:**

To describe head computed tomography (CT) findings in neonates with congenital Zika virus infection confirmed in cerebrospinal fluid.

**Materials and Methods:**

This was a study of 16 newborn infants who exhibited abnormal head CT findings during an outbreak of Zika virus infection. Those infants had the following features: brain imaging suggestive of congenital infection; brain calcifications and negative results on tests for other main infectious causes of primary microcephaly, namely toxoplasmosis, cytomegalovirus, rubella, and HIV; positivity for Zika virus on IgM antibody capture enzyme-linked immunosorbent assay in cerebrospinal fluid.

**Results:**

Decreased brain volume was observed in 13 (81.2%) of the infants. All of the infants showed cortico-subcortical calcifications, mainly located in the frontal lobe. In 15 neonates (93.7%), ventriculomegaly was observed. Colpocephaly was a common finding, being observed in 10 patients (62.5%). A prominent occipital bone was identified in 9 patients (56.2%).

**Conclusion:**

Our study proves that Zika virus infection can cause congenital brain damage, with or without microcephaly. Some predominant head CT findings in neonates with congenital Zika virus infection, although not pathognomonic, are strongly suggestive of a pattern.

## INTRODUCTION

In October 2015, the Pan American Health Organization/World Health Organization (PAHO/WHO) reported the detection of an abnormal increase in the number of cases of microcephaly at public and private health care facilities in the Brazilian state of Pernambuco, located in the northeastern region of the country^(^^1^^)^.

Until an outbreak in French Polynesia in the 2013-2014 period, the disease caused by infection with the Zika virus (ZIKV) was recognized only as a mild disease. During that outbreak, the incidence of Guillain-Barré syndrome was 20 times higher than expected^(^^2^^)^. Similarly, the appearance of ZIKV in the Americas, beginning in 2015, coincided with a dramatic increase in reported cases of microcephaly. Epidemiological data suggest that cases of microcephaly in Brazil are associated with the introduction of ZIKV. Some evidence of vertical transmission of ZIKV was also reported. On January 2016, the Brazilian National Ministry of Health reported the detection of the ZIKV genome by means of the reverse-transcription polymerase chain reaction (RT-PCR) technique in four cases of congenital malformation in the northeastern Brazilian state of Rio Grande do Norte. The cases corresponded to two abortions and two full-term newborns that died within the first 24 hours of life. Tissue samples from both neonates were also positive for ZIKV by immunohistochemistry^(^^3^^)^. Next, the ZIKV genome was detected in samples of amniotic fluid from two pregnant women in the state of Paraíba, also in northeastern Brazil, whose fetuses were diagnosed with microcephaly by obstetric ultrasound^(^^4^^)^.

The WHO Emergency Committee reported that the recent increase in cases of microcephaly and other neurological disorders reported in Brazil follows a pattern similar to that of French Polynesia in 2014 and is a public health emergency of international importance^(^^5^^)^. The PAHO/WHO reiterates recommendations related to ZIKV surveillance, including monitoring of neurological syndromes and congenital anomalies. Infants who meet the microcephaly criteria should be evaluated by teams of qualified physicians to determine the extent of neurological damage and other possible abnormalities. Additional (laboratory and radiological) studies should be performed in accordance with local protocols, including the research of other causes of microcephaly, especially those requiring treatment, such as congenital syphilis, cytomegalovirus, and toxoplasmosis^(^^6^^)^.

Non-contrast-enhanced computed tomography (CT) of the head is part of the clinical and epidemiological protocol for the investigation of cases of microcephaly in Pernambuco, as instituted by the Pernambuco State Department of Health in partnership with the National Ministry of Health and the other institutions involved in the response to this event^(^^7^^)^.

This article aims to describe the head CT findings in newborns with congenital ZIKV infection confirmed in cerebrospinal fluid.

## MATERIALS AND METHODS

This was a case series of 16 newborns under investigation for microcephaly, who presented cranioencephalic changes on non-contrast-enhanced CT of the head, conducted as part of the protocol established by the National Ministry of Health during an outbreak of congenital ZIKV infection, from October 2015 to February 2016. The study was approved by the Research Ethics Committee of Hospital Otávio de Freitas (Reference no. 51275815.3.0000.5200).

We conducted a descriptive (retrospective and prospective) study by reviewing the medical records of patients diagnosed with congenital ZIKV infection who underwent non-contrast-enhanced CT of the head at Barão de Lucena Hospital during the microcephaly epidemic in Brazil.

According to the protocol of the Brazilian National Department of Public Health, all children with suspected microcephaly should be referred to one of the pediatric infectious diseases departments for continued investigation. The first reference criterion of the protocol was a head circumference below 33 cm. As of December 2, 2015, the criterion was reduced to 32 cm for infants with a gestational age of 37 weeks or more and at least two standard deviations below the mean for age and gender on the Fenton growth chart for preterm infants.

This case series describes 16 patients diagnosed with congenital ZIKV infection, all of whom met the following inclusion criteria: images suggestive of brain infection; a completely negative investigation for the other major infectious causes of primary microcephaly and calcifications in the brain parenchyma (toxoplasmosis, cytomegalovirus, rubella, and HIV); and immunoglobulin M (IgM) enzyme-linked immunosorbent assay (ELISA) positivity for ZIKV in cerebrospinal fluid. Positive serology for syphilis did not constitute an exclusion criterion.

All of the examinations were performed on a multislice CT scanner and analyzed by the same radiologist. The images were considered suggestive of congenital infection if calcifications were present. A standard form was used in order to collect demographic and clinical data, including whether mothers remembered having had a skin rash during pregnancy.

The main congenital infections that cause cerebral calcifications and microcephaly-cytomegalovirus, toxoplasmosis, rubella, and HIV-were investigated through IgM and IgG serological tests of the mother and the newborn. If cytomegalovirus IgG was present in both, PCR was performed in the urine. Patients with known causes of microcephaly other than ZIKV were excluded from the study. Cerebrospinal fluid samples were collected in the first week of life of the newborn and were tested for ZIKV by the IgM ELISA method, following the protocol of the Centers for Disease Control and Prevention, as described by Martin et al.^(^^8^^)^.

Microcephaly is an important sign, although it is not present in all cases of congenital Zika syndrome, and head circumference normal for gestational age and gender is not an exclusion factor. According to the Fetal International and Newborn Growth Consortium for the 21st Century (Intergrowth-21st), microcephaly is defined as a head circumference two standard deviations below the mean for gestational age and gender, and severe microcephaly is defined as a head circumference three standard deviations below the mean for gestational age and gender. Weight was evaluated at birth and classified, on a case-by-case basis, as below or above the norm for gestational age and gender, according to the Intergrowth-21st curve.

## RESULTS

At this writing, 61 patients have undergone a CT scan of the head, according to the protocol of the State of Pernambuco, at the Barão de Lucena Hospital. The images of the skull and brain presented findings suggestive of congenital infections in 24 patients, 16 of whom met the inclusion criteria. Two of the infants had concomitant congenital syphilis.

We reported head CT results for 16 newborns (9 males and 7 females). The gestational age at birth ranged from 31 weeks to 40 weeks (three infants being premature), the birth weight ranged from 810 g to 3840 g, and the head circumference ranged from 23 cm to 33 cm. All of the newborns had a birth weight appropriate for gestational age, and 12 had microcephaly.

Cerebral volume reduction was seen in 13 (81.2%) of the newborns, and those patients also presented some type of malformation of cortical development. The volume reduction of the brain parenchyma, as determined by qualitative analysis, was mild in two newborns (15.4%), moderate in three (23.0%), and severe in eight (61.6%).

**Figure 1:**
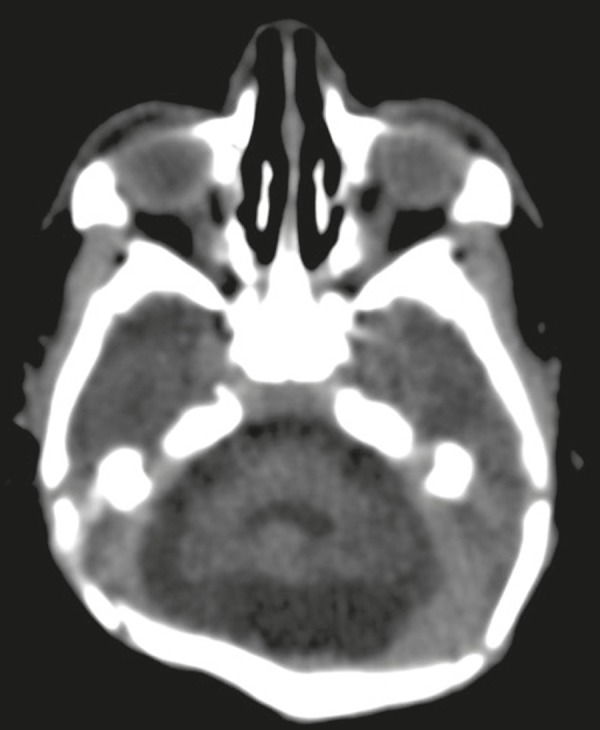
Non-contrast-enhanced CT images of the brain. Calcification patterns at the cortico-subcortical junction: punctate (**A,B**) and coarse (**C,D**). Signs of cerebral volume reduction associated with malformation of cortical development and ventriculomegaly (**A,C,D**) and colpocephaly (**A,C**).

Central nervous system (CNS) calcifications were observed in all patients, being punctate in nine (56.2%), and coarse in seven (43.7%). All of the infants showed cortical-subcortical calcifications. Calcifications were also seen in the basal ganglia in two infants (12.5%), in the thalamus in four (25.0%), and in the brainstem in two (12.5%). As can be seen in Figure 1, the cortical-subcortical calcifications were located mainly in the frontal lobe (in 100% of the cases) and parietal lobes (in 68.7%), as well as, less frequently, in the occipital lobe (in 50%) and temporal lobes (in 43.7%).

**Figure 2:**
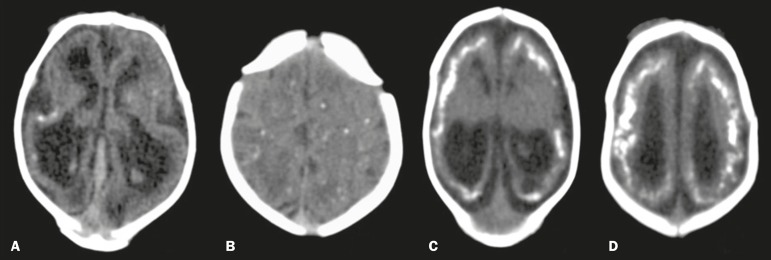
Non-contrast-enhanced CT scan showing cerebellar hypoplasia.

**Figure 3:**
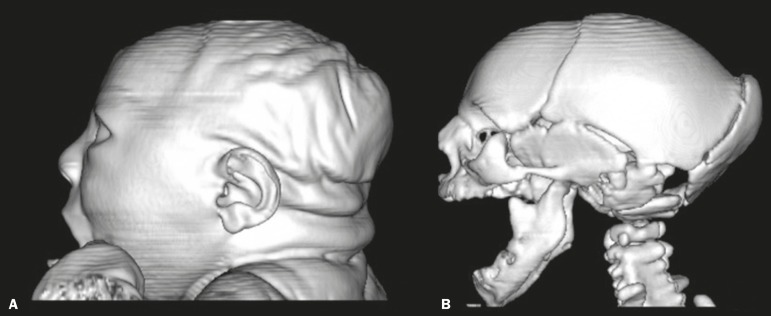
Three-dimensional reconstruction of head CT. **A:** Severe microcephaly with scalp folds. **B:** Prominent occipital bone and overlapping sutures.

Ventriculomegaly was observed in 15 newborns (93.7%), being supratentorial in 10 (66.7%) and global in 5 (33.3%). Colpocephaly was a common finding, seen in 10 patients (62.5%), and was associated with the parallel appearance of the lateral ventricles (50%). Cerebellar hypoplasia was seen in three patients (18.7%), as shown in Figure 2. A prominent occipital bone, as depicted in Figure 3, was identified in nine newborns (56.2%). Two infants (12.5%) had periventricular encephalomalacia.

Two patients were diagnosed with concomitant congenital syphilis, presenting cortical and periventricular calcifications, one of them also presenting open-lip schizencephaly.

## DISCUSSION

The present study describes the head CT findings in 16 confirmed cases of ZIKV infection implicated in congenital microcephaly. In three studies found in the literature describing these changes^(^^9^^-^^11^^)^, the majority of cases were attributed to presumed, although unconfirmed, congenital ZIKV infection (Table 1). The presumptive criteria for ZIKV infection used in these studies were clinical and epidemiological aspects, as well as negative serology for other congenital infections, especially the infections that make up the Toxoplasmosis, Other (syphilis, varicella-zoster, parvovirus B19), Rubella, Cytomegalovirus, and Herpes (TORCH) group. Congenital microcephaly is a descriptive term that means small head at birth and is associated with numerous disorders of various causes. A recent study reported that ZIKV efficiently infects and replicates in the forebrain (human neural progenitor cells).These cells, when infected, can release new virus particles, leading to a disseminated infection, up to 90% of the cells being ZIKV positive^(^^12^^)^. We find it interesting that ZIKV appears to be less infectious for more developed neural cells, which suggests that fetal brains are probably much more susceptible to ZIKV than are adult brains. Another study described the preference of ZIKV for radial glial cells and developing brain stem cells, whose depletion leads to microcephaly^(^^13^^)^. As described in other congenital infections, we assume that more severe cases of microcephaly are associated with early congenital infection. We have used the term "cerebral volume reduction" rather than "cerebral atrophy", because there are no widely accepted hypotheses to explain the pathophysiology and the main ones that exist involve cell apoptosis without necrosis or destruction.

**Table 1:** Comparison among studies.

	Findings											
Article	Number of patients with Zika virus infection	Cerebral volume reduction	Malformation of cortical development	Type of calcification		Location of calcifications						
						Cortico-subcortical junction	Basal ganglia	Thalamus	Brainstem and cerebellum	Ventriculomegaly	Occipital bone prominence	Cerebellar hypoplasia
				Punctate	Coarse							
Hazin et al.^(^^9^^)^	7 confirmed	Not	23 (100%)	72-100%	-	53-86%	57-65%	39-43%	0	23 (100%)	Not	17 (74%)
	16 presumed	mentioned									mentioned	
Aragão et al.^(^^10^^)^	6 confirmed	20 (91%)	21 (95%)	Majority	-	23 (100%)	13 (59%)	0	Brainstem	19 (86%)	7 (30%)	11 (50%)
	17 presumed								8 (35%)			
									Cerebellum			
									11 (50%)			
Cavalheiro et al.^(^^11^^)^	13 presumed	13 (100%)	13 (100%)	0	12 (92.3%)	12 (92.3%)	12 (92.3%)	0	0	13 (100%)	13 (100%)	0
This article	16 confirmed	13 (81.2%)	13 (81.2%)	9 (56.2%)	7 (43.7%)	16 (100%)	2 (12.5%)	4 (25%)	2 (12.5%)	15 (93.7%)	9 (56.2%)	3 (18.7%)

Cerebral volume reduction was seen in 13 (81.2%) of the infants, all of whom also presented some type of malformation of cortical development. Aragão et al.^(^^10^^)^ and Cava lheiro et al.^(^^11^^)^, respectively, evaluated 23 and 13 children with microcephaly and obtained similar results. The finding of malformation of cortical development was also consistent with Hazin et al.^(^^9^^)^, however, this study does not mention the cerebral volume reduction. We should emphasize the fact that, in our study, four (25%) of the newborns did not have microcephaly at birth, according to gestational age and gender. However, in those same newborns, head CT revealed cerebral calcifications and mild ventriculomegaly, suggesting the complexity of the spectrum of the syndrome.

Calcifications in the CNS are a common finding in congenital infections, including TORCH group infections. In TORCH group infections, CNS calcifications are predominantly periventricular, when caused by cytomegalovirus, or diffuse and can attack the basal nuclei, white matter, and cortex. Our study demonstrated a cortico-subcortical calcification pattern in all cases, especially punctate calcifications (in 56.2%) and, to a lesser degree, coarse calcifications (in 43.7%). Cortico-subcortical calcifications were also described in all patients in the study conducted by Aragão et al.^(^^10^^)^ and were the most prevalent type of calcifications in the studies conducted by Hazin et al.^(^^9^^)^ and Cavalheiro et al.^(^^11^^)^, who identified such calcifications in 53-86% and 92.3% of cases, respectively. The distribution of calcifications in the present study was also concordant with the results of Aragão et al.^(^^10^^)^ and Hazin et al.^(^^9^^)^, the calcifications in our patient sample being most commonly located in the frontal lobe (in 100% of cases) and parietal lobes (in 68.7%), followed by the occipital lobe (in 50.0%) and temporal lobes (in 43.7%). To a lesser degree, calcifications were also observed in the basal ganglia (in 12.5%), thalamus (in 25.0%), and brainstem (in 12.5%). Aragão et al.^(^^10^^)^ also described cerebellar calcifications, which were not observed in our study, in 11 cases (50.0%). We suggest that the high prevalence of CNS calcifications at the cortico-subcortical junction, identified primarily in the frontal lobes, in all studies, represents a marked sign in the pattern of involvement of congenital Zika syndrome.

Ventriculomegaly is another finding of high prevalence in previous studies, occurring in 100% of the cases evaluated by Hazin et al.^(^^9^^)^ and Cavalheiro et al.^(^^11^^)^, as well as in 86% of the cases evaluated by Aragão et al.^(^^10^^)^. In agreement with the findings of those studies, ventricular dilatation was identified in 93.7% of our cases, being mainly supratentorial, particularly in the posterior portions of the lateral ventricles (colpocephaly). We observed ventriculomegaly *ex vacuo*, defined as a compensatory increase in cerebrospinal fluid volume and widening of the ventricles, due to a loss of brain volume. The presence of colpocephaly might be associated with agenesis of the corpus callosum, although that finding was not analyzed, due to the technical limitation of head CT, magnetic resonance imaging being the more appropriate choice for this purpose.

Cerebellar hypoplasia was observed in only three (18.7%) of our patients. The prevalence was lower than that reported by Hazin et al.^(^^9^^)^ and Aragão et al.^(^^10^^)^, who identified cerebellar hypoplasia in 74% and 50% of cases, respectively. This divergence can be explained by the fact that head CT is limited in its capacity to evaluate the posterior fossa.

A prominent occipital bone, identified in nine of our patients, can be associated with the fetal brain disruption sequence, which is characterized by severe microcephaly, overlapping sutures, scalp wrinkles, and marked neurological impairment, reflecting significant intrauterine brain damage. Aragão et al.^(^^10^^)^ and Cavalheiro et al.^(^^11^^)^ reported that finding in 7 (30%) and 13 (100%) of the infants evaluated, respectively.

We decided not to exclude the two infants with syphilis and congenital Zika syndrome, in order to assess whether the imaging findings were more pronounced in individuals with concomitant infection. CT scans of their heads presented cortical and periventricular calcifications, in addition to other findings similar to those seen in the patients with ZIKV infection alone. This pattern of calcification is not a typical finding in isolated congenital syphilis, being more characteristic of congenital cytomegalovirus infection. Aragão et al.^(^^10^^)^ and Cavalheiro et al.^(^^11^^)^ identified this distribution in 10 (45%) and 1 (7.7%) of cases of presumed ZIKV infection, respectively. These discordant incidences could be explained by the difficulty of accurately determining the actual location of calcifications in cases in which the parenchyma is very thin.

We would like to emphasize that, in our study, we selected patients with positive findings on CT, which does not exclude the possibility of normal CT findings in patients with congenital ZIKV infection, and this should be studied in the near future.

Most of the CNS changes described in the present study, such as malformations of cortical development, calcifications, ventriculomegaly, and cerebellar hypoplasia, are not pathognomonic of congenital ZIKV infection and can occur in other conditions, especially in congenital TORCH group infections. However, in relation to calcifications, the pattern of distribution at the cortico-subcortical junction, especially in the frontal lobes, is not a common finding in TORCH group infections and could represent a specific pattern of congenital Zika syndrome. Therefore, we recommend that ZIKV congenital infection be systematically considered, together with TORCH group infections, in the differential diagnosis of these conditions, especially in cases of CNS calcification.

## CONCLUSION

Some predominant tomographic findings on head CT scans of newborns with congenital Zika syndrome, although not pathognomonic, are strongly suggestive of a pattern: cerebral volume reduction, malformation of cortical development, calcifications (predominantly at the cortico-subcortical junction, in the frontal lobes), ventriculomegaly (especially with colpocephaly), and prominence of the occipital bone.

In view of the increase in the number of cases of congenital ZIKV infection, the possibility of global dissemination of the virus, and of outbreaks in the world, it is necessary to know the most common tomographic findings.

## References

[1] Pan American Health Organization, World Health Organization Epidemiological alert: increase of microcephaly in the northeast of
Brazil - 17 November 2015.

[2] Musso D, Nilles EJ, Cao-Lormeau VM (2014). Rapid spread of emerging Zika virus in the Pacific
area. Clin Microbiol Infect.

[3] Brasil, Ministério da Saúde, Centro de operações de emergências em
saúde pública sobre microcefalias Informe epidemiológico nº 08 - Monitoramento dos casos de
microcefalia no Brasil.

[4] Calvet G, Aguiar RS, Melo ASO (2016). Detection and sequencing of Zika virus from amniotic fluid of
fetuses with microcephaly in Brazil: a case study. Lancet Infect Dis.

[5] World Health Organization WHO statement on the first meeting of the International Health
Regulations (2005) (IHR 2005) Emergency Committee on Zika virus and observed
increase in neurological disorders and neonatal malformations.

[6] Pan American Health Organization, World Health Organization Epidemiological update: neurological syndrome, congenital anomalies and
Zika virus infection. 17 January 2016.

[7] Secretaria Estadual de Saúde de Pernambuco Protocolo clínico e epidemiológico - microcefalia.
Versão nº 02..

[8] Martin DA, Muth DA, Brown T (2000). Standardization of immunoglobulin M capture enzyme-linked
immunosorbent assays for routine diagnosis of arboviral
infections. J Clin Microbiol.

[9] Hazin AN, Poretti A, Cruz DDCS (2016). Computed tomographic findings in microcephaly associated with
Zika virus. N Engl J Med.

[10] Aragão MFV, van der Linden V, Brainer-Lima AM (2016). Clinical features and neuroimaging (CT and MRI) findings in
presumed Zika virus related congenital infection and microcephaly:
retrospective case series study. BMJ.

[11] Cavalheiro S, Lopez A, Serra S (2016). Microcephaly and Zika virus: neonatal neuroradiological
aspects. Childs Nerv Syst.

[12] Guo J (2016). Studies using IPS cells support a possible link between ZIKA and
microcephaly. Cell Biosci.

[13] (2016). Zika research shifts into high gear. Cell.

